# Fine‐mapping of HLA class I and class II genes identified two independent novel variants associated with nasopharyngeal carcinoma susceptibility

**DOI:** 10.1002/cam4.1838

**Published:** 2018-10-30

**Authors:** Tong‐Min Wang, Ting Zhou, Yong‐Qiao He, Wen‐Qiong Xue, Jiang‐Bo Zhang, Xiao‐Hui Zheng, Xi‐Zhao Li, Shao‐Dan Zhang, Yi‐Xin Zeng, Wei‐Hua Jia

**Affiliations:** ^1^ State Key Laboratory of Oncology in South China Collaborative Innovation Center for Cancer Medicine Guangdong Key Laboratory of Nasopharyngeal Carcinoma Diagnosis and Therapy Sun Yat‐sen University Cancer Center Guangzhou China; ^2^ School of Public Health Sun Yat‐Sen University Guangzhou China; ^3^ Cancer Center of Guangzhou Medical University Guangzhou China

**Keywords:** fine‐mapping, human leukocyte antigen, nasopharyngeal carcinoma, susceptibility

## Abstract

**Background:**

Several genome‐wide association studies (GWASs) have identified strong associations between genetic variants in the human leukocyte antigen (HLA) region and nasopharyngeal carcinoma (NPC). However, given the complex LD pattern in this region, the causal variants and the underlying mechanism of how genetic variants in HLA contribute to NPC development is yet to be understood.

**Methods:**

To systematically characterize the HLA variants and their relationship to NPC susceptibility, we fine‐mapped the HLA genes based on the GWAS data of 1583 NPC cases and 972 healthy controls, using SNP2HLA with the Pan‐Asian panel as references. Stepwise conditional regression was used to identify independent association loci.

**Results:**

Interestingly, the most significant association was the presence of Gln in HLA‐A amino acid position 62 (OR = 0.57, *P* = 1.41 × 10^−16^). The G allele of rs2894207 located between HLA‐B and HLA‐C showed protective effect of NPC development (OR = 0.52, *P* = 2.23 × 10^−13^). Additionally, amino acid Phe‐67 located in the peptide‐binding pocket of HLA‐DRB1 was identified as a novel functional variant with OR = 0.64 and *P* = 9.64 × 10^−11^. Another novel variant, Glu‐45 in HLA‐B pocket B, conferred a protective effect on NPC susceptibility (OR = 0.64, *P* = 5.23 × 10^−8^). These four variants explained 2.07% of the phenotypic variance for NPC risk.

**Conclusion:**

In summary, by fine‐mapping the HLA region in south Chinese population, we reported additional loci missed in the GWAS studies and provided a better understanding of the relationship between HLA and NPC susceptibility.

## 1 INTRODUCTION

Nasopharyngeal carcinoma (NPC) is an epithelial squamous cell carcinoma with a distinct incidence around the world. In endemic regions including southern China and other Southeast Asian countries, the incidence rate reached 25‐50 per 100 000 people, which is remarkably higher than that in the rest of the world.^1^, ^2^, ^3^, ^4^ In addition to the unbalanced geographic distribution, familial clustering of NPC was often observed, and approximately 10% of NPC patients have familial history,^5^ indicating the genetic contributions to NPC susceptibility. Apart from host genetics, environmental exposures and Epstein‐Barr virus (EBV) infection also play important roles in NPC development. Tobacco smoking and salted fish consumption are associated with increased NPC risk.^6^, ^7^, ^8^ Several long‐term prospective studies showed that people who carried elevated VCA‐IgA, EA‐IgA, or DNase may have a 20‐30 fold increase in NPC risk in endemic areas,^9^, ^10^ and a recent study showed that EBV plasma DNA was a useful biomarker for early detection of NPC.^11^

Among the host genetic markers that have been associated with NPC, strong, and consistent association signals have been shown in the human leukocyte antigen (HLA) region. In the genome‐wide association studies (GWASs) across southern China, Taiwan, and Malaysia, a total of 15 SNPs in the HLA region were identified and confirmed as association markers of NPC risk.^12^, ^13^, ^14^, ^15^ Proteins encoded by HLA class I and class II genes affect host responses to EBV infection through viral antigen presentation. Variations in amino acid residues at specific positions may influence the antigen‐presenting process and facilitate tumor cell evasion of host immune surveillance.^16^ Recently, a GWAS study fine‐mapped the HLA class I genes and detected several functional amino acids variants in the HLA‐A, HLA‐B, and HLA‐C loci.^14^ In addition, another GWAS study in Malaysian Chinese used high‐resolution fine‐mapping on HLA‐A and identified some variants in the antigen peptide‐binding groove and T‐cell receptor binding site.^13^

In our previous GWAS in southern Chinese, three NPC protective SNPs were detected in this region including one located in 4 kb upstream of HLA‐A, one in the intergenic region of HLA‐B and HLA‐C and one located between the HLA‐DRB1 and DQA1 loci.^12^ However, interpretations of these results are difficult because in the current opinion, the reported SNPs are proxy for association signals without direct biological function. In addition, the identification of driving variants is problematic owing to the complicated LD pattern of this gene‐dense region. To further study these loci, we conducted a fine‐mapping study on our preexisting GWAS data using SNP2HLA,^17^ an HLA‐specific imputation tool to systematically explore the potential causal variants in the whole HLA region that drives the predisposition to nasopharyngeal carcinoma in southern Chinese population.

## 2 MATERIALS AND METHODS

### 2.1 Subjects, genotyping, and quality control

Subjects were from a large GWAS study and were fully described elsewhere.^12^ In brief, a total of 1615 cases were recruited from Sun Yat‐sen University Cancer Center and were pathologically diagnosed according to the WHO classification. During the same period, 1025 healthy controls from 21 municipalities in Guangdong Province were recruited from physical examination centers of hospitals in Guangdong and had no history of malignancy. All cases and controls in this study are self‐reported as Guangdong Chinese and lived in Guangdong Province at the time of the study. Age (±5 years), gender, geographic region, and ethnicity are matched by frequency between cases and controls.

Genotyping of the 2640 GWAS samples was performed using Illumina Human610‐Quad BeadChips (620 900 SNPs). We followed the previously described quality control procedures 12 and removed SNPs with call rates < 95%, SNPs with minor allele frequency (MAF) <3% and SNPs with deviation from Hardy‐Weinberg equilibrium in controls (*P* < 10^−6^). In addition, samples with genotype call rates <96%, duplicates or relatives identified by identity‐by‐descent analysis and population outliers detected by principal component analysis were also excluded. After quality control, a total of 465 618 SNPs in 1583 cases and 972 controls were included in this study.

### 2.2 Imputation of HLA variants

The study design and analysis workflow are shown in Figure S1. First, 2602 SNPs within the entire HLA region from 29 to 34 Mb on chromosome 6 (NCBI Build 36) were extracted from the GWAS data described above. Using these data, we imputed two‐digit and four‐digit classical HLA alleles, amino acid polymorphisms in HLA‐A, HLA‐B, HLA‐C, and HLA‐DRB1, HLA‐DQA1, HLA‐DQB1, HLA‐DPA1, and HLA‐DPB1 as well as the un‐genotyped SNPs in this region using SNP2HLA software.^17^ The Pan‐Asian reference panel was used in our analysis, which includes Han Chinese population, Southeast Asian Malaysian population, Tamil Indian and Japanese population.^18^, ^19^ After imputation, quality control was conducted again to exclude variants with low imputation quality. Here, we removed variants with INFO<0.5 or MAF<0.01. The INFO score was defined by the ratio of the observed variance in dosage to the expected variance under Hardy‐Weinberg equilibrium. Finally, a total of 6124 variants, which consisted of 5144 SNPs, 832 amino acid polymorphisms and 148 two‐digit and four‐digit classical HLA alleles remained in our further analysis.

### 2.3 Statistical analysis

All association analyses were conducted by R and plink (v1.07). There were four types of HLA variants in our imputed data set including bi‐allelic SNPs, bi‐allelic classical HLA alleles, bi‐allelic amino acid polymorphisms, and multi‐allelic amino acid polymorphisms. The effects of all variants were assumed to be additive on a log (Odds) scale and represented by the allele dosages (posterior genotype probability). For each bi‐allelic variant, a traditional logistic regression model adjusted with age and gender was used to test its effect. For each multi‐allelic amino acid polymorphism consisting of m alleles, two types of test were conducted. First, the effect of each allele (Present vs Absent) in a specific multi‐allelic position was tested using traditional logistic regression as dichotomous variable. Additionally, to investigate the overall effect of the multi‐allelic site, an omnibus *P* value was calculated based on the log‐likelihood ratio test by comparing the likelihood of the null model *L*_0_ (2) to the likelihood of the full model *L*_1_ (1).^20^ Here, the full model is represented by

(1) $$\log\left({\text{Odds}}_{k}\right)=\beta_{0}+\sum_{i=1}^{m-1}\beta_{1,i}x_{i,k}+\beta_{2}{\text{Age}}_{k}+\beta_{3}{\text{Gender}}_{k}$$

where β_0_ is the regression intercept, β_1,*i*_ is the effect size of dosage allele *i* among the m alleles and β_2_, β_3_ are the effect sizes of age and gender, respectively. The null model is represented by

(2) $$\log\left({\text{Odds}}_{k}\right)=\beta_{0}+\beta_{2}{\text{Age}}_{k}+\beta_{3}{\text{Gender}}_{k}$$

and the test statistic with m‐1 degree of freedom was written as

(3) $$D=-2\text{ln}\frac{L_{0}}{L_{1}},\;D∼\chi_{\left(m-1\right)}^{2}.$$

After imputation and post‐imputation quality control, 6124 variants were considered in our study. We set the significance threshold of the association study by applying Bonferroni correction (8.16 × 10^−6^, 0.05/6124).

Stepwise conditional regression analysis was performed to explore the potential independent driving variants of NPC. In each step, the most significant variant was added as a covariate in the model until no remaining variants can reach the significant threshold (*P* = 8.16 × 10^−6^). Then, a permutation test was also used to test the identified variants and confirm the result of our discovery. The binary phenotypes were resampled 10 000 times to generate permutation test statistics. Permutation *P* values were calculated by comparing the observed test statistic and the permutation test statistic.^21^ We tested the cumulative or additive effect of the candidate variants by counting the number of protective alleles at each locus and used the Cochran‐Armitage trend test to detect the trend effect.^22^, ^23^ The cumulative ORs for subjects carrying different copies of protective alleles were estimated by comparing them with those carrying none of these protective alleles. LD statistics (*D*′ and *r* ^2^) were calculated by Haploview version 4.2 to explore the relationship between variants.^24^ Haplotype estimation was performed by the R package Haplostats.^25^

### 2.4 Variance explained by identified variants

The variance of phenotype explained by specific groups of genetic loci was estimated using a fixed effects model as described previously.^20^, ^26^, ^27^ The four identified variants in our study, seven SNPs reported in our previous GWAS study on NPC were used to calculate the respective variances.^12^ We used the NPC five‐year prevalence 28 to transform the estimated heritability from an observed scale to a liability scale.

## 3 RESULTS

### 3.1 Association analysis for single variants in HLA region

After the association analysis was performed for each variant in a logistic regression model, a total of 357 variants showed significant associations with NPC, including 270 SNPs, 17 bi‐allelic and 59 multi‐allelic amino acid polymorphisms and 11 two‐digit and four‐digit HLA classical alleles (Table S1).

In the previous GWAS study on NPC, a total of 15 SNPs in the HLA region were reported.^12^, ^13^, ^14^, ^15^ In this study, we replicated three SNPs in GABBR1, two SNPs in HLA‐A, two SNPs in HCG and one SNP in HLA‐B/C with association significance at GWAS level (*P* < 5 × 10^−8^). In addition, two risk alleles in the HLA‐F locus (rs3129055 and rs9258122) detected by Tse et al^15^ also showed significance with *P* values at the level of 10^−5^. Four SNPs in HLA‐A were not imputed because none of them were included in the Pan‐Asian reference panel (Table S2).

Among the 148 2‐digit and 4‐digit HLA classical alleles included in our analysis, 11 of them (located in HLA‐A, B, DRB1, and DQB1 loci) were significantly associated with NPC. Our result further confirmed the previous discovery of association between classical alleles in class I and class II HLA genes and NPC susceptibility (Table S3). To further explore the combination effect of the significant alleles, we estimated their haplotypes using the significantly associated HLA alleles above and found a total of four haplotypes constructed by class I and class II genes that were associated with NPC susceptibility. Two NPC protective haplotypes, HLA‐A*11—HLA‐B*13—HLA‐DQB1*03 and HLA‐A*11:01—HLA‐B*13:01—HLA‐DQB1*03:01 showed significance with *P*_trend _< 10^−20^. A stronger protective effect was observed in individuals carrying at least one classic allele in their HLA‐A*11—HLA‐B*13—HLA‐DQB1*03 or HLA‐A*11:01—HLA‐B*13:01—HLA‐DQB1*03:01 haplotypes. Decreased ORs were observed from 0.71 to 0.30. Additionally, association was also detected on two risk HLA‐A—HLA‐B haplotypes with an increased odds ratio from 1.27 to 1.64 and *P*_trend _= 1.13 × 10^−9^ (Table S4).

We detected 42 amino acid polymorphisms in class I genes (HLA‐A and HLA‐B) and 12 in class II genes (HLA‐DQB1 and HLA‐DRB1) as significant association signals in our study (Table S5). Association signals were led by a previously identified amino acid HLA‐A Gln‐62 (OR = 0.54, *P* = 2.81 × 10^−22^). Previous studies have shown that HLA‐A Gln‐62 marked the HLA‐A*11:01 allele together with several HLA‐A amino acids nearby in the same LD block.^12^, ^13^ In addition, some amino acids in HLA‐B also showed significant with NPC susceptibility, led by the amino acid Leu at position −16 and 116 in high LD with each other (*D*′ = 0.928, *r* ^2 ^= 0.58). Although HLA‐C Trp‐156 was detected as an association signal in Tang's research,^14^ it did not reach the significance level after Bonferroni correction (OR = 0.63, *P* = 6.59 × 10^−5^). For the amino acids in HLA class II genes, we discovered twelve amino acid polymorphisms in HLA‐DRB1 and HLA‐DQB1 associated with NPC susceptibility (Table S5), led by HLA‐DQB1 Glu‐45 (OR = 0.62, *P* = 6.58 × 10^−11^). LD analysis showed that polymorphism of this amino acid was in high LD not only with other significant amino acid variants in this region (Figure S2) but also with classical alleles including HLA‐DQB1*03:01 (*D*′ = 0.996, *r* ^2 ^= 0.983), HLA‐DQB1*03 (*D*′ = 0.998, *r* ^2 ^= 0.337), and HLA‐DRB1*11:01 (*D*′ = 0.986, *r* ^2 ^= 0.256).

### 3.2 Stepwise conditional analysis detected four potential driving variants

**Figure 1:**
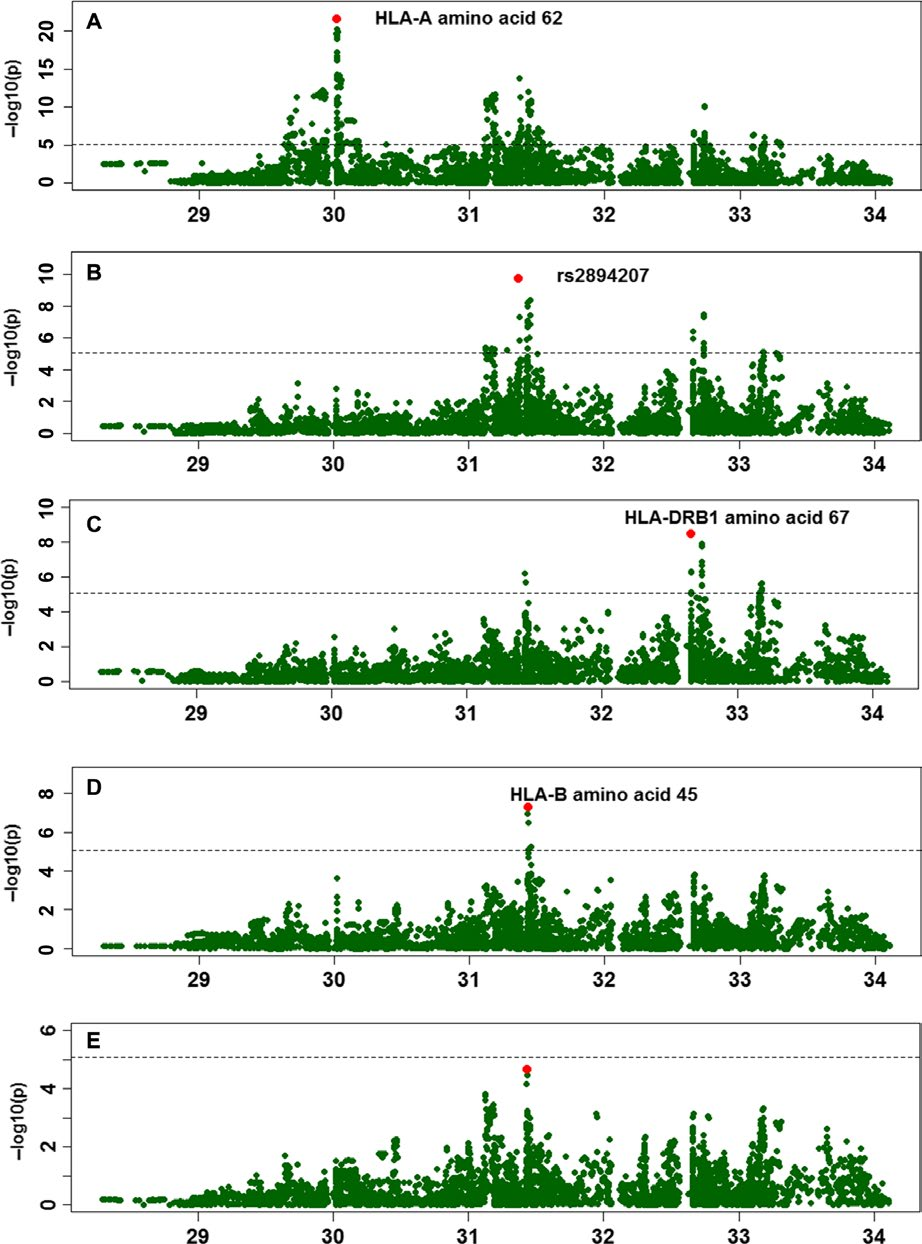
Stepwise conditional regression result of HLA loci independently associated with NPC susceptibility. Imputed allelic dosage (between 0 and 2) of HLA variants including SNPs, amino acid polymorphisms, and classic alleles were tested. In each panel, each point represented ‐log_10_(*P* value) of the variants, and the dashed line marked the Bonferroni *P* value (*P *<* *8.16 × 10^−6^). A, The strongest association was amino acid Gln‐62 in the HLA‐A locus (OR = 0.54, *P* = 2.81 × 10^−22^). B, After adjustment for age, gender, and HLA‐A Gln‐62, the most significant signal was SNP rs2894207 (OR = 0.58, *P* = 1.73 × 10^−10^). C, When age, gender, HLA‐A Gln‐62, and rs2894207 were used as covariates, we observed HLA‐DRB1 Phe‐67 to be the most significant variant (OR = 0.67, *P* = 3.3 × 10^−9^). D, After adjustment for age, gender, HLA‐A Gln‐62, rs2894207, and HLA‐DRB1 Phe‐67, we detected HLA‐B Glu‐45 as the fourth most significant variant (OR = 0.64, *P* = 5.23 × 10^−8^). E, When conditioning on age, gender, HLA‐A Gln‐62, rs2894207, HLA‐DRB1 Phe‐67, and HLA‐B Glu‐45, no remaining variants in the HLA region reached the Bonferroni threshold (Table S6)

Since the HLA region has a complex LD pattern, we identified the independent variants that may drive NPC susceptibility using stepwise conditional analysis. In each step, the most significant variant was included in the next model as a covariate until no variant reached the significance level (*P* < 8.16 × 10^−6^). When conditioned on age and gender, the most significant association signal was the HLA‐A amino acid Gln variant at position 62 (OR = 0.54, *P* = 2.81 × 10^−22^; Figure 1A, Table S6). After adjustment for age, gender, and HLA‐A Gln‐62, rs2894207, a SNP reported in our previous GWAS study, showed the leading significant signal among the remaining variants (OR = 0.58, *P* = 1.73 × 10^−10^) (Figure 1B, Table S6). When age, gender, HLA‐A Gln‐62, and rs2894207 were used as covariates, we observed a third independent variant, HLA‐DRB1 Phe‐67 (OR = 0.67, *P* = 3.3 × 10^−9^) (Figure 1C, Table S6). After adjustment for age, gender, HLA‐A Gln‐62, rs2894207, and HLA‐DRB1 Phe‐67, we identified HLA‐B Glu‐45 as the fourth most significant variant (OR = 0.64, *P* = 5.23 × 10^−8^) (Figure 1D, Table S6). When conditioning on age, gender, HLA‐A Gln‐62, rs2894207, HLA‐DRB1 Phe‐67, and HLA‐B Glu‐45, no remaining variants in the HLA region reached the Bonferroni threshold (Figure 1E, Table S6). An LD plot confirmed that these four variants were statistically independent of each other. All of the four variants were further confirmed by permutation testing (Table S6 and S7).

After conducting stepwise conditional analysis, we built a multivariate NPC susceptibility model which incorporated four independent protective HLA variants. All variants in the model showed protective effect (Tables 1 and S7) on NPC. Individuals carrying HLA‐A Gln‐62 (OR = 0.57, *P* = 1.41 × 10^−16^), the rs2894207G allele (OR = 0.52, *P* = 2.23 × 10^−13^), HLA‐DRB1 Phe‐67 (OR = 0.64, *P* = 9.64 × 10^−11^), or HLA‐B Glu‐45 (OR = 0.64, *P* = 5.23 × 10^−8^) showed lower NPC risk compared with those carrying the reference alleles.

**Table 1:** Associations of HLA variants with NPC susceptibility

HLA variants	Effect^a^	Reference^b^	EAF^c^		OR (95% CI)^d^	*P* d
			Cases (n = 1583)	Control (n = 972)		
HLA‐A amino acid position 62 (Gln)	Present	Absent	26.56%	39.71%	0.57 (0.5,0.65)	1.41 × 10^−16^
rs2894207	G	A	9.76%	17.64%	0.52 (0.44,0.62)	2.23 × 10^−13^
HLA‐DRB1 amino acid position 67 (Phe)	Present	Absent	31.11%	38.17%	0.64 (0.56,0.74)	9.64 × 10^−11^
HLA‐B amino acid position 45 (Glu)	Present	Absent	14.88%	19.39%	0.64 (0.54,0.75)	5.23 × 10^−8^

Effective alleles, for HLA‐A amino acid polymorphisms in site 62, the effective allele was the presence of glutamine (Gln); for HLA‐DRB1 amino acid polymorphisms in site 67, the effective allele was the presence of phenylalanine (Phe); for HLA‐B amino acid polymorphisms in site 45, the effective allele was the presence of glutamic acid (Glu).

Reference alleles, the absence of the effective allele was treated as the reference allele here.

Effective allele frequency in cases and controls.

ORs and *P* values were obtained from a multivariable model constructed by the four potential driving variants and covariates including age and gender.

### 3.3 LD analysis of four driving variants and the previously reported variants

**Figure 2:**
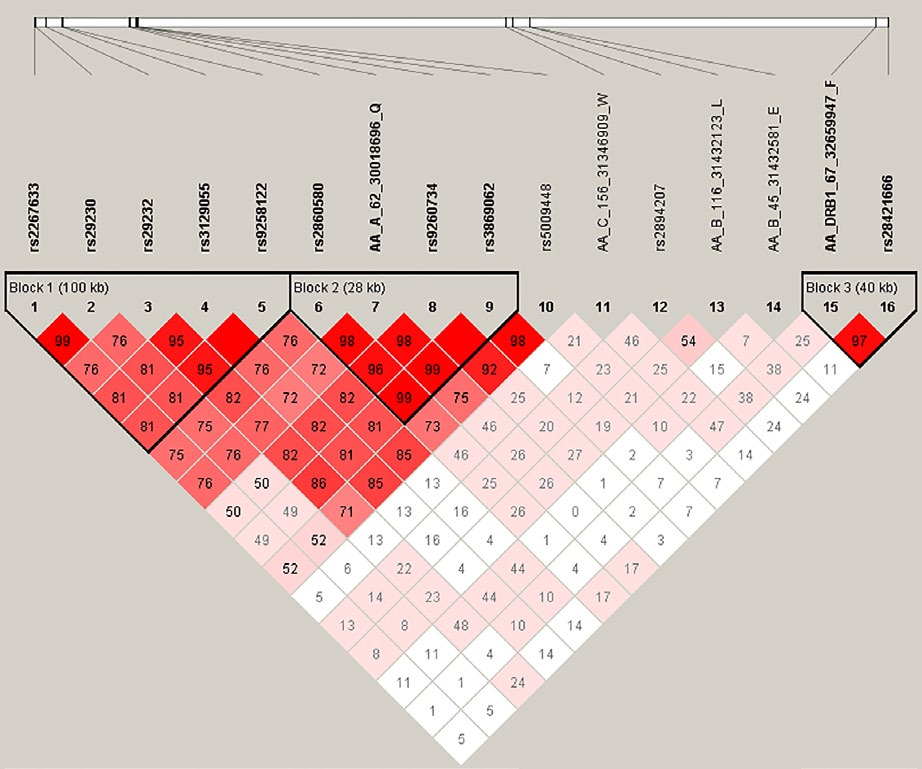
LD plot of the four potential driving variants and the previously reported variants

Several SNPs and functional amino acid polymorphisms in the HLA region have been detected as susceptibility loci for NPC.^13^, ^14^ To explore their relationship with our four identified variants, a systematic LD analysis was performed (Table S8, Figure 2). Strong LD was shown in HLA‐A Gln‐62 with SNPs in HLA‐A and its adjacent SNPs, including rs2860580 (*D*′ = 0.985, *r* ^2 ^= 0.941), which was reported in our previous GWAS study. The identified intergenic SNP rs2894207 located between HLA‐B and HLA‐C was in medium LD with amino acid HLA‐B Leu‐116 (*D*′ = 0.545, *r* ^2 ^= 0.286) and HLA‐C Trp‐156 (*D*′ = 0.468, *r* ^2 ^= 0.103) but in low LD with HLA‐B Glu‐45 (*D*′ = 0.158, *r* ^2 ^= 0.001). The amino acid in class II gene HLA‐DRB1 Phe‐67 was associated with rs28421666 (*D*′ = 0.977, *r* ^2 ^= 0.247) and when conditioning on HLA‐DRB1 Phe‐67, the association *P* value of rs28421666 increased dramatically (OR = 0.76, *P* = 0.00716). Low LD was observed for HLA‐B Glu‐45 with any other reported variants.

### 3.4 Cumulative effect of four variants

Taking into account the four potential driving variants with protective effects on NPC, we moved on to explore their cumulative effect. Analysis of a combination of the four variants provided a stronger cumulative association with NPC (*P*_trend _= 1.03 × 10^−35^) than any individual variant (Tables 2 and S7). Compared with subjects without any of the protective variants, individuals with one to four protective variants showed lower NPC risk with ORs of 0.63, 0.33, 0.19, and 0.11.

**Table 2:** Cumulative effect of potential driving variants

No. of effective variants[Link]	Frequency in cases	Frequency in controls	Odd ratio (95% CI)[Link]	*P* [Link]
0	215 (14.02%)	54 (5.7%)	Reference	
1	599 (39.05%)	238 (25.13%)	0.63 (0.45,0.88)	8.62 × 10^−3^
2	547 (35.66%)	412 (43.51%)	0.33 (0.24,0.46)	1.33 × 10^−11^
3	154 (10.04%)	200 (21.12%)	0.19 (0.13,0.28)	1.07 × 10^−19^
4	19 (1.24%)	43 (4.54%)	0.11 (0.06,0.21)	5.05 × 10^−14^
Trend				1.03 × 10^−35^

The number of effective variants was counted in each sample.

ORs and *P* values were obtained by comparing the samples carrying a specific number of effective variants with those carrying no effective variant.

### 3.5 Variance explained by four variants

Based on the four identified variants in our study and those detected SNPs located in HLA and non‐HLA regions in our previous study, we estimated the genetic variance explained (*h* ^2^) with a liability threshold model, assuming an NPC prevalence of 0.000078.^28^ The four identified variants in our study explained *h* ^2 ^= 2.07% on a liability scale, whereas the associated HLA and non‐HLA SNPs in our previous GWAS study explained 1.73% and 0.40%, respectively (Table 3). The four variants in this study can explain 72.89% of the phenotypic variance owing to genetic variants.

**Table 3:** Heritability estimates for the four identified variants and significantly associated SNPs in our previous GWAS study

Model[Link]	*h* ^2^	*h* ^2^
	Liability scale[Link]	Observed scale
Four identified HLA variants	2.07%	8.09%
Three HLA SNPs identified in our GWAS	1.73%	7.24%
Four non‐HLA SNPs identified in our GWAS	0.40%	2.01%
Combined	2.84%	10.37%

Genetic variants used for estimating heritability, including four variants identified in our stepwise conditional regression, three HLA SNPs and four non‐HLA SNPs detected in our previous GWAS study.

Five‐year prevalence of NPC (0.000078) was used for the heritability estimate on the liability scale.^28^

## 4 DISCUSSION

Human leukocyte antigens (HLA) have been proposed as important host genetic factors associated with NPC, given their central role in viral antigen presentation to the immune system.^29^ An association between HLA genes and NPC was first proposed by Simons and colleagues.^30^ Since then, the association between HLA and NPC has been further confirmed by over one hundred candidate association studies. In recent years, four independent GWASs have consistently reported SNPs in the HLA region to have the strongest association signals with NPC. Given the crucial role of HLA genes, fine‐mapping of the HLA locus is beneficial to further explore the functional variants involved in NPC development. In this study, we imputed the HLA region by an HLA‐specific imputation tool SNP2HLA, using the Pan‐Asian panel as references and detected four potential driving variants that were independently associated with NPC susceptibility by stepwise conditional regression analysis. We identified two novel amino acid polymorphisms, HLA‐DRB1 amino acid Phe in site 67 and HLA‐B amino acid Glu in site 45 associated with NPC risks. These two loci, combined with the other two known loci, HLA‐A amino acid Gln in site 62 and rs2894207 (located in intergenic region of HLA‐B and HLA‐C), made a cumulative contribution to NPC risk and explained 2.07% of NPC susceptibility.

The imputation method for fine‐mapping has been successfully applied to other immune‐related disease such as celiac disease, Graves’ disease and type 1 diabetes.^20^, ^26^, ^27^ Although we did not genotype the HLA region directly, we fine‐mapped this region and repeated associations of some important classic alleles associated with NPC, such as protective alleles HLA‐A*11:01, HLA‐B*13:01 and risk alleles HLA‐A*2, HLA‐B*46:01. Additionally, we also replicated some important amino acid polymorphisms in HLA class I genes.

The NPC association signals were driven by the presence of glutamine at HLA‐A position 62. This variant has been identified in two NPC GWASs 13, 14 and is now repeated in our imputation data. It marked the NPC protective classic allele HLA‐A*11:01 and was highly correlated with rs2860580, the association signal observed in our previous GWAS study. HLA‐A amino acid site 62 is located in exon 2, which encode the alpha 1 domain. Polymorphisms within this region as well as in exon 3 are responsible for the peptide‐binding specificity of each class one molecule.

HLA‐B Glu‐45, located in pocket B of the HLA‐B peptide‐binding groove, is one of the functional variants that was newly identified in this study. In a previous fine‐mapping study of NPC, another amino acid Leu‐116 in HLA‐B pocket F, was identified as a protective allele in NPC.^14^ Although they were close in physical position, a low correlation was shown in the LD analysis. HLA‐B plays an important part in the body's immune response to viral attack. A recent GWAS study of HIV immune control in different population demonstrated that specific amino acids in the HLA‐B peptide‐binding groove played roles in modulating durable control of HIV infection.^31^

The signals of NPC association in HLA class II genes were represented by the presence of phenylalanine at HLA‐DRB1 position 67, which is a newly identified functional variant associated with NPC in our study. This variant is located in exon 2 of the HLA‐DRB1 locus, which plays a central role in the immune system by presenting peptides derived from extracellular proteins. A previous study has identified an amino acid signature at positions 26, 67, 71, and 74 in the peptide‐binding pocket of DR β‐chain to be strongly associated with type 1 diabetes (T1D) and autoimmune thyroid disease (AITD).^32^ In our previous GWAS study, rs28421666 in the DR/DQ region was identified as one of the NPC association loci. When conditioning on HLA‐DRB1 Phe‐67, the association *P* value of rs28421666 increased dramatically, suggesting that the previously identified signal in our GWAS study may be driven by this functional variant. Analysis also showed that Phe‐67 in DRB1 was in high LD with classic alleles HLA‐DRB1*11:01, HLA‐DQB1*03, and HLA‐DQB1*03:01. While associations were detected for all of them in our study, the systematic study of HLA class I and class II alleles in Taiwanese did not observe their associations with NPC.^33^ Therefore, Phe‐67 in DRB1 may be a new functional variant playing an important role in NPC development.

In summary, we fine‐mapped the HLA region, further exploring the GWAS data of NPC by imputation, and identified four protective variants that may influence NPC susceptibility independently. Our study is beneficial for a deeper understanding of the relationship between host genetic factors and NPC predisposition. Apart from previously identified variants, we reported additional loci that were missed in the GWAS studies and fine‐mapping studies, which may further explain the complex HLA association with NPC in the southern Chinese population. Further studies on the functional basis are warranted to uncover the underlying mechanism of how HLA variations contribute to NPC predisposition.

## CONFLICT OF INTEREST

The authors made no disclosures.

## References

[1] Jia WH , Huang QH , Liao J , et al. Trends in incidence and mortality of nasopharyngeal carcinoma over a 20‐25 year period (1978/1983‐2002) in Sihui and Cangwu counties in southern China. BMC Cancer. 2006;6:178. 16822324 10.1186/1471-2407-6-178 PMC1557527

[2] Torre LA , Bray F , Siegel RL , Ferlay J , Lortet‐Tieulent J , Jemal A . Global cancer statistics, 2012. CA Cancer J Clin. 2015;65(2):87‐108. 25651787 10.3322/caac.21262

[3] Yu MC , Yuan JM . Epidemiology of nasopharyngeal carcinoma. Semin Cancer Biol. 2002;12(6):421‐429. 12450728 10.1016/s1044579x02000858

[4] Tang LL , Chen WQ , Xue WQ , et al. Global trends in incidence and mortality of nasopharyngeal carcinoma. Cancer Lett. 2016;374(1):22‐30. 26828135 10.1016/j.canlet.2016.01.040

[5] Jia WH , Feng BJ , Xu ZL , et al. Familial risk and clustering of nasopharyngeal carcinoma in Guangdong, China. Cancer. 2004;101(2):363‐369. 15241835 10.1002/cncr.20372

[6] Chang ET , Liu Z , Hildesheim A , et al. Active and passive smoking and risk of nasopharyngeal carcinoma: a population‐based case‐control study in Southern China. Am J Epidemiol. 2017;185(12):1272‐1280. 28459936 10.1093/aje/kwx018 PMC5860561

[7] Xue WQ , Qin HD , Ruan HL , Shugart YY , Jia WH . Quantitative association of tobacco smoking with the risk of nasopharyngeal carcinoma: a comprehensive meta‐analysis of studies conducted between 1979 and 2011. Am J Epidemiol. 2013;178(3):325‐338. 23785114 10.1093/aje/kws479 PMC3727336

[8] Lau HY , Leung CM , Chan YH , et al. Secular trends of salted fish consumption and nasopharyngeal carcinoma: a multi‐jurisdiction ecological study in 8 regions from 3 continents. BMC Cancer. 2013;13:298. 23782497 10.1186/1471-2407-13-298 PMC3729410

[9] Chien YC , Chen JY , Liu MY , et al. Serologic markers of Epstein‐Barr virus infection and nasopharyngeal carcinoma in Taiwanese men. N Engl J Med. 2001;345(26):1877‐1882. 11756578 10.1056/NEJMoa011610

[10] Cao SM , Liu Z , Jia WH , et al. Fluctuations of epstein‐barr virus serological antibodies and risk for nasopharyngeal carcinoma: a prospective screening study with a 20‐year follow‐up. PLoS ONE. 2011;6(4):e19100. 21544243 10.1371/journal.pone.0019100 PMC3081347

[11] Chan KCA , Woo JKS , King A , et al. Analysis of plasma epstein‐barr virus DNA to screen for nasopharyngeal cancer. N Engl J Med. 2017;377(6):513‐522. 28792880 10.1056/NEJMoa1701717

[12] Bei JX , Li Y , Jia WH , et al. A genome‐wide association study of nasopharyngeal carcinoma identifies three new susceptibility loci. Nat Genet. 2010;42(7):599‐603. 20512145 10.1038/ng.601

[13] Chin YM , Mushiroda T , Takahashi A , et al. HLA‐A SNPs and amino acid variants are associated with nasopharyngeal carcinoma in Malaysian Chinese. Int J Cancer. 2015;136(3):678‐687. 24947555 10.1002/ijc.29035

[14] Tang M , Lautenberger JA , Gao X , et al. The principal genetic determinants for nasopharyngeal carcinoma in China involve the HLA class I antigen recognition groove. PLoS Genet. 2012;8(11):e1003103. 23209447 10.1371/journal.pgen.1003103 PMC3510037

[15] Tse KP , Su WH , Chang KP , et al. Genome‐wide association study reveals multiple nasopharyngeal carcinoma‐associated loci within the HLA region at chromosome 6p21.3. Am J Hum Genet 2009;85(2):194‐203. 19664746 10.1016/j.ajhg.2009.07.007 PMC2725267

[16] Young LS , Dawson CW . Epstein‐Barr virus and nasopharyngeal carcinoma. Chin J Cancer. 2014;33(12):581‐590. 25418193 10.5732/cjc.014.10197 PMC4308653

[17] Jia X , Han B , Onengut‐Gumuscu S , et al. Imputing amino acid polymorphisms in human leukocyte antigens. PLoS ONE. 2013;8(6):e64683. 23762245 10.1371/journal.pone.0064683 PMC3675122

[18] Okada Y , Kim K , Han B , et al. Risk for ACPA‐positive rheumatoid arthritis is driven by shared HLA amino acid polymorphisms in Asian and European populations. Hum Mol Genet. 2014;23(25):6916‐6926. 25070946 10.1093/hmg/ddu387 PMC4245039

[19] Pillai NE , Okada Y , Saw WY , et al. Predicting HLA alleles from high‐resolution SNP data in three Southeast Asian populations. Hum Mol Genet. 2014;23(16):4443‐4451. 24698974 10.1093/hmg/ddu149

[20] Okada Y , Momozawa Y , Ashikawa K , et al. Construction of a population‐specific HLA imputation reference panel and its application to Graves’ disease risk in Japanese. Nat Genet. 2015;47(7):798‐802. 26029868 10.1038/ng.3310

[21] Ernst MD . Permutation methods: a basis for exact inference. Stat Sci. 2004;19(4):676‐685.

[22] Armitage P . Tests for linear trends in proportions and frequencies. Biometrics. 1955;11(3):375‐386.

[23] Cochran WG . Some methods for strengthening the common X2 tests. Biometrics. 1954;10(4):417‐451.

[24] Barrett JC , Fry B , Maller J , Daly MJ . Haploview: analysis and visualization of LD and haplotype maps. Bioinformatics. 2005;21(2):263‐265. 15297300 10.1093/bioinformatics/bth457

[25] Schaid DJ , Rowland CM , Tines DE , Jacobson RM , Poland GA . Score tests for association between traits and haplotypes when linkage phase is ambiguous. Am J Hum Genet. 2002;70(2):425‐434. 11791212 10.1086/338688 PMC384917

[26] Gutierrez‐Achury J , Zhernakova A , Pulit SL , et al. Fine mapping in the MHC region accounts for 18% additional genetic risk for celiac disease. Nat Genet. 2015;47(6):577‐578. 25894500 10.1038/ng.3268 PMC4449296

[27] Hu XL , Deutsch AJ , Lenz TL , et al. Additive and interaction effects at three amino acid positions in HLA‐DQ and HLA‐DR molecules drive type 1 diabetes risk. Nat Genet 2015;47(8):898‐+. 26168013 10.1038/ng.3353 PMC4930791

[28] Dai J , Shen W , Wen W , et al. Estimation of heritability for nine common cancers using data from genome‐wide association studies in Chinese population. Int J Cancer. 2017;140(2):329‐336. 27668986 10.1002/ijc.30447 PMC5536238

[29] Su WH , Hildesheim A , Chang YS . Human leukocyte antigens and epstein‐barr virus‐associated nasopharyngeal carcinoma: old associations offer new clues into the role of immunity in infection‐associated cancers. Front Oncol. 2013;3:299. 24367763 10.3389/fonc.2013.00299 PMC3856645

[30] Simons MJ , Wee GB , Day NE , Morris PJ , Shanmuga K , Dethe GB . Immunogenetic Aspects of Nasopharyngeal Carcinoma .1. Differences in Hl‐a antigen profiles between patients and control groups. Int J Cancer 1974;13(1):122‐134. 4131857 10.1002/ijc.2910130114

[31] Pereyra F , Jia X , McLaren PJ , et al. The major genetic determinants of HIV‐1 control affect HLA class I peptide presentation. Science. 2010;330(6010):1551‐1557. 21051598 10.1126/science.1195271 PMC3235490

[32] Menconi F , Osman R , Monti MC , Greenberg DA , Concepcion ES , Tomer Y . Shared molecular amino acid signature in the HLA‐DR peptide binding pocket predisposes to both autoimmune diabetes and thyroiditis. Proc Natl Acad Sci U S A. 2010;107(39):16899‐16903. 20837527 10.1073/pnas.1009511107 PMC2947869

[33] Hildesheim A , Apple RJ , Chen CJ , et al. Association of HLA class I and II alleles and extended haplotypes with nasopharyngeal carcinoma in Taiwan. J Natl Cancer Inst. 2002;94(23):1780‐1789. 12464650 10.1093/jnci/94.23.1780

