# Supplementary figures and images for: Fine‐mapping of HLA class I and class II genes identified two independent novel variants associated with nasopharyngeal carcinoma susceptibility
Source: Cancer Med. 2018 Oct 30;7(12):6308–16. doi: 10.1002/cam4.1838 (PMC6308056; doi:10.1002/cam4.1838)

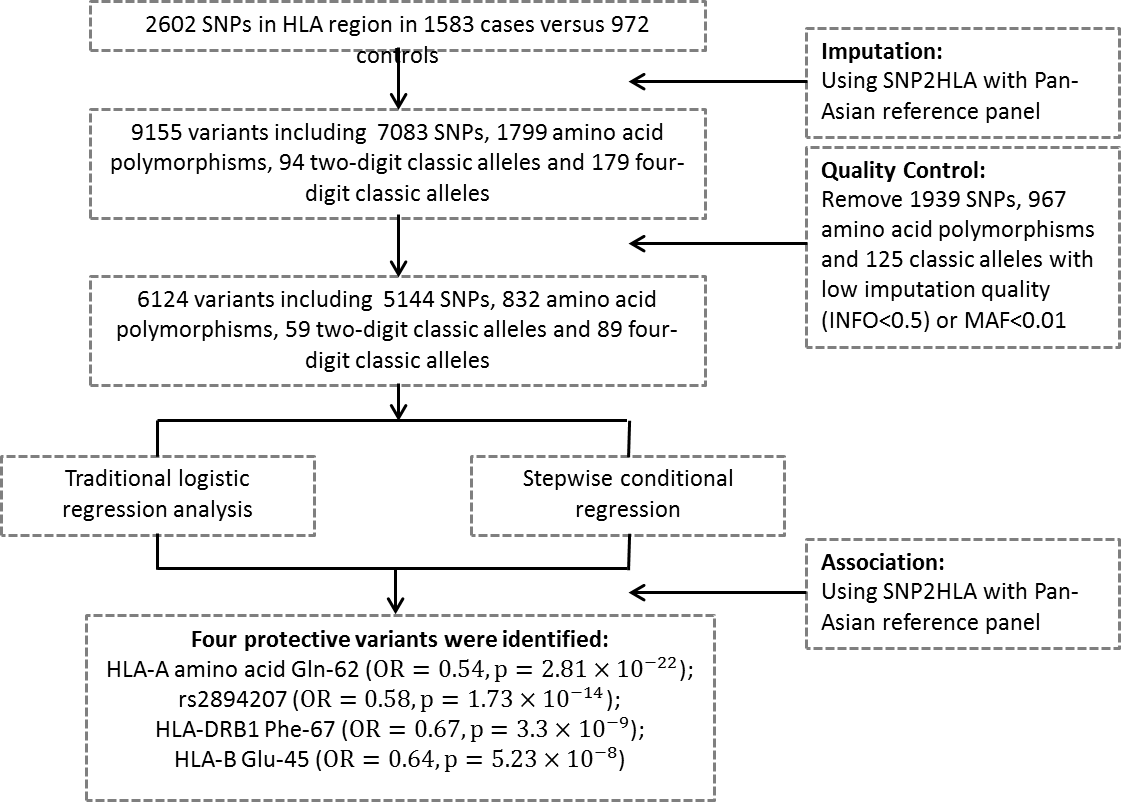

Supplement: Supplementary file 1 [file CAM4-7-6308-s001.tif]

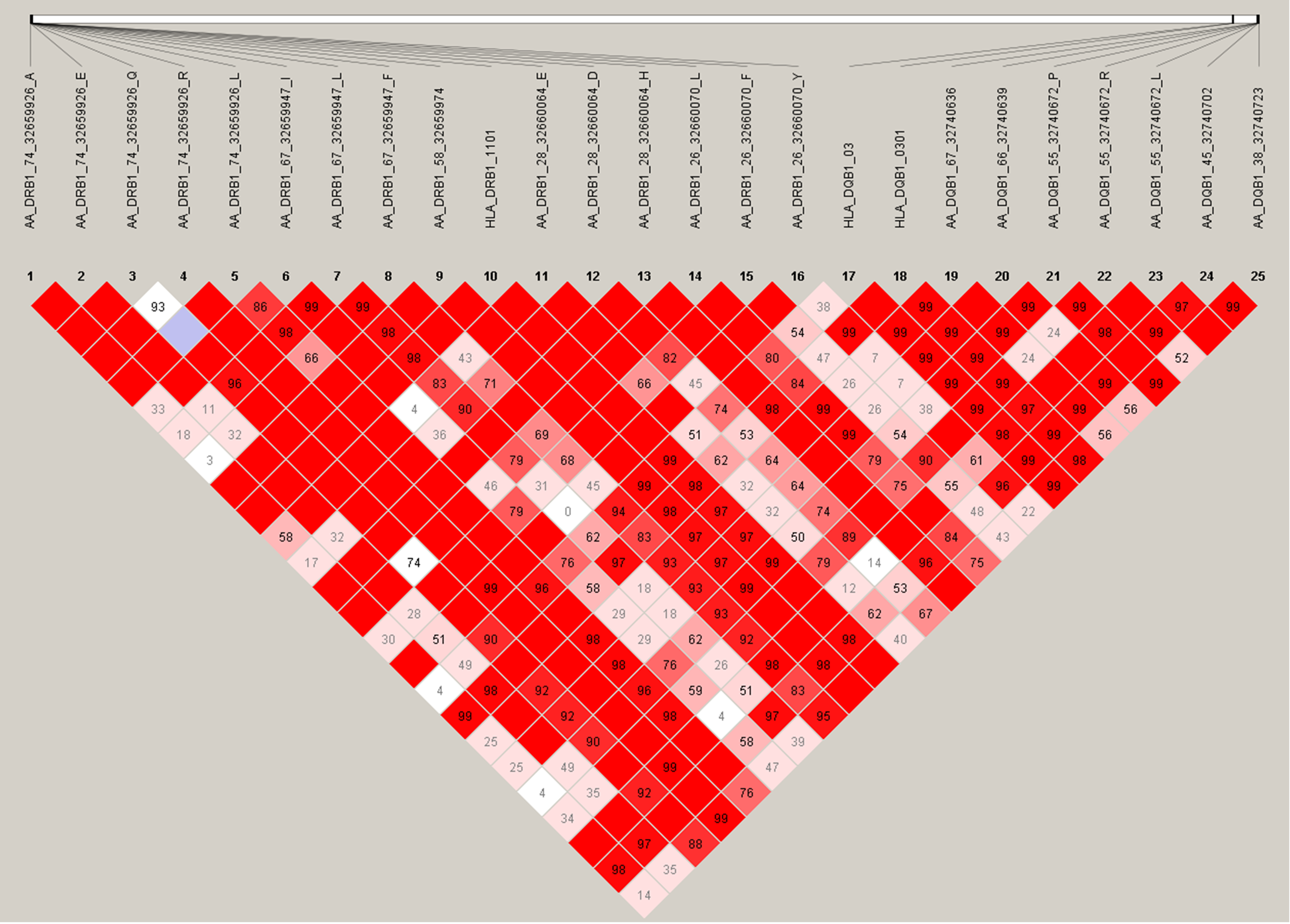

Supplement: Supplementary file 2 [file CAM4-7-6308-s002.tif]
