# Supplementary material for: Fine‐mapping of HLA class I and class II genes identified two independent novel variants associated with nasopharyngeal carcinoma susceptibility
Source: Cancer Med. 2018 Oct 30;7(12):6308–16. doi: 10.1002/cam4.1838 (PMC6308056; doi:10.1002/cam4.1838)
Supplement: Supplementary file 4 [file CAM4-7-6308-s004.docx]

Supplementary Figure S1 Study design and work flow.

Supplementary Figure S2 LD plot of significant HLA-DQ/DR amino acid polymorphisms and classic alleles.

Supplementary Table S2 Summary of previously discovered SNPs in our GWAS data.

| **SNP** | **Nearby Gene** | **Position^a^** | **EFF^b^** | **REF^c^** | **FEQ** | **EAF^d^** | | **OR(95%CI)^e^** | **P^e^** | **Previous GWAS Studies^f^** |
| --- | --- | --- | --- | --- | --- | --- | --- | --- | --- | --- |
|  |  |  |  |  |  | **Cases** | **Controls** |  |  |  |
| rs2267633 | GABBR1 | 29678820 | G | A | 0.220 | 0.193 | 0.265 | 0.66(0.57,0.75) | 2.24E-09 | MingZhongTang,et al, 2012; Tse,et al, 2009 |
| rs29230 | GABBR1 | 29684372 | G | A | 0.220 | 0.193 | 0.265 | 0.66(0.57,0.75) | 2.19E-09 | MingZhongTang,et al, 2012; Tse,et al, 2009 |
| rs29232 | GABBR1 | 29719410 | A | G | 0.489 | 0.527 | 0.427 | 1.5(1.34,1.69) | 4.41E-12 | Tse,et al, 2009 |
| rs3129055 | HLA-F | 29778240 | G | A | 0.348 | 0.369 | 0.314 | 1.27(1.13,1.43) | 8.42E-05 | Tse,et al, 2009 |
| rs9258122 | HLA-F | 29779719 | A | G | 0.348 | 0.369 | 0.314 | 1.27(1.13,1.43) | 8.42E-05 | Tse,et al, 2009 |
| rs2860580 | HLA-A | 30014670 | A | G | 0.309 | 0.262 | 0.386 | 0.55(0.49,0.63) | 2.25E-20 | Bei JX,et al. 2010 |
| rs41545520^g^ | HLA-A | 30026078 | - | - | - | - | - | - | - | Yoon-Ming Chin,et al, 2015 |
| rs417162^g^ | HLA-A | 30028515 | - | - | - | - | - | - | - | MingZhongTang,et al, 2012 |
| rs2517713^g^ | HLA-A | 30018243 | - | - | - | - | - | - | - | Tse,et al, 2009; MingZhongTang,et al, 2012 |
| rs2975042^g^ | HLA-A | 30024484 | - | - | - | - | - | - | - | Tse,et al, 2009 |
| rs3869062 | HLA-A | 30042870 | G | A | 0.241 | 0.206 | 0.298 | 0.59(0.52,0.68) | 7.12E-14 | Yoon-Ming Chin,et al, 2015; Tse,et al, 2009 |
| rs9260734 | HCG9 | 30040645 | A | G | 0.257 | 0.220 | 0.318 | 0.59(0.52,0.68) | 6.87E-15 | MingZhongTang,et al, 2012; Tse,et al, 2009 |
| rs5009448 | HCG9 | 30048467 | A | G | 0.301 | 0.263 | 0.362 | 0.63(0.55,0.71) | 2.27E-13 | MingZhongTang,et al, 2012; Tse,et al, 2009 |
| rs2894207 | HLA-B/C | 31371730 | G | A | 0.131 | 0.098 | 0.181 | 0.52(0.44,0.62) | 1.73E-14 | Bei JX,et al. 2010 |
| rs28421666 | HLA-DQ/DR | 32700715 | G | A | 0.117 | 0.098 | 0.145 | 0.66(0.56,0.78) | 2.22E-06 | Bei JX,et al. 2010 |

**^a^** Physical position based on hg 18

**^b^** Effective allele

**^c^** Reference allele

**^d^** Effective allele frequency in cases and controls

**^e^** Odds ratio and p value in logistic regression model adjusted with age and gender

**^f^** Previously reports: MingZhongTang,et al, 2012 [[1](#_ENREF_1)]; Tse,et al, 2009 [[2](#_ENREF_2)]; Bei JX,et al. 2010 [[3](#_ENREF_3)]; Yoon-Ming Chin,et al, 2015 [[4](#_ENREF_4)]

**^g^** SNPs not included in Pan-Asian panel

Supplementary Table S3 Summary of the significantly associated classic alleles.

| **Classic allele** | **EFF^a^** | **REF^b^** | **FRQ** | **EAF^c^** | | **OR(95%CI)^d^** | **P^d^** |
| --- | --- | --- | --- | --- | --- | --- | --- |
|  |  |  |  | **Cases** | **Controls** |  |  |
| HLA-A*02 | P | A | 0.376 | 0.403 | 0.332 | 1.37(1.21,1.55) | 5.08E-07 |
| HLA-A*11 | P | A | 0.279 | 0.237 | 0.348 | 0.56(0.49,0.64) | 5.23E-18 |
| HLA-A*11:01 | P | A | 0.233 | 0.195 | 0.296 | 0.56(0.49,0.64) | 6.65E-17 |
| HLA-B*13 | P | A | 0.076 | 0.061 | 0.101 | 0.57(0.46,0.7) | 1.62E-07 |
| HLA-B*13:01 | P | A | 0.065 | 0.051 | 0.087 | 0.56(0.45,0.7) | 6.20E-07 |
| HLA-B*46 | P | A | 0.192 | 0.213 | 0.158 | 1.46(1.25,1.7) | 1.44E-06 |
| HLA-B*46:01 | P | A | 0.192 | 0.213 | 0.158 | 1.46(1.25,1.7) | 1.44E-06 |
| HLA-DRB1*11 | P | A | 0.049 | 0.038 | 0.067 | 0.49(0.37,0.64) | 4.00E-07 |
| HLA-DRB1*11:01 | P | A | 0.049 | 0.038 | 0.067 | 0.49(0.37,0.64) | 4.06E-07 |
| HLA-DQB1*03 | P | A | 0.434 | 0.406 | 0.478 | 0.73(0.65,0.82) | 2.26E-07 |
| HLA-DQB1*03:01 | P | A | 0.213 | 0.185 | 0.258 | 0.62(0.54,0.72) | 9.68E-11 |

**^a^** Effective allele, P: Present

**^b^** Reference allele, A: Absence

**^c^** Effective allele frequency in cases and controls

**^d^** Odds ratio and p value in logistic regression model adjusted with age and gender

Supplementary Table S4 Summary of significantly associated haplotypes constructed by significant HLA classic alleles.

| **Effect** | **Haplotype** | **# of alleles present in haplotype^a^** | **EAF^b^** | | **OR (95%CI)^c^** | **P^c^** |
| --- | --- | --- | --- | --- | --- | --- |
|  |  |  | **Case** | **Control** |  |  |
| Protective | HLA-A*11--HLA-B*13--HLA-DQB1*03 | 0 | 45.67% | 33.95% | Reference |  |
|  |  | 1 | 40.90% | 42.85% | 0.71(0.63,0.81) | 1.27E-07 |
|  |  | 2 | 12.92% | 21.97% | 0.44(0.37,0.52) | 2.25E-23 |
|  |  | 3 | 0.51% | 1.23% | 0.30(0.16,0.58) | 2.30E-04 |
|  |  | trend test ^d^ |  |  |  | 3.26E-25 |
| Protective | HLA-A*11:01--HLA-B*13:01—HLA-DQB1*03:01 | 0 | 66.77% | 52.42% | Reference |  |
|  |  | 1 | 25.90% | 34.10% | 0.60(0.53,0.68) | 1.26E-15 |
|  |  | 2 | 7.08% | 12.81% | 0.43(0.36,0.53) | 2.07E-17 |
|  |  | 3 | 0.25% | 0.67% | 0.30(0.12,0.72) | 8.62E-03 |
|  |  | trend test ^d^ |  |  |  | 9.34E-27 |
| Risk | HLA-A*02--HLA-B*46 | 0 | 54.11% | 61.99% | Reference |  |
|  |  | 1 | 29.72% | 26.75% | 1.27(1.12,1.45) | 3.07E-04 |
|  |  | 2 | 16.17% | 11.27% | 1.64(1.38,1.96) | 2.46E-08 |
|  |  | trend test ^d^ |  |  |  | 1.13E-09 |
| Risk | HLA-A*02--HLA-B*4601 | 0 | 54.11% | 61.99% | Reference |  |
|  |  | 1 | 29.72% | 26.75% | 1.27(1.12,1.45) | 3.07E-04 |
|  |  | 2 | 16.17% | 11.27% | 1.64(1.38,1.96) | 2.46E-08 |
|  |  | trend test ^d^ |  |  |  | 1.13E-09 |

**^a^** Number of alleles that presented in the haplotypes.

**^b^** Effective allele frequency in cases and controls.

**^c^** Odds ratios and p values were calculated by allele test. We compared individuals carrying specific number of alleles in their haplotypes with those without any effect allele in their haplotypes.

**^d^** Cochran-Armitage trend test was used to calculate the trend test p value.

Supplementary Table S7 Associations for each identified variants.

| **Step** | **HLA variants ^a^** | **EAF ^b^** | | **OR(95% CI)^c^** | **P ^c^** |
| --- | --- | --- | --- | --- | --- |
|  |  | **Case (N=1583)** | **Control (N=972)** |  |  |
| Step I | HLA-A amino acid position Gln-62 |  |  |  |  |
|  | Non-effect/Non-effect | 840(53.06%) | 345(35.49%) | Reference |  |
|  | Effect/Non-effect | 645(40.75%) | 482(49.59%) | 0.55(0.46,0.65) | 1.01E-11 |
|  | Effect/Effect | 98(6.19%) | 145(14.92%) | 0.28(0.21,0.37) | 1.24E-19 |
|  | Trend ^d^ |  |  |  | 1.69E-23 |
|  | Permutation ^e^ |  |  |  | <1.0E-04 |
| Step II | rs2894207 |  |  |  |  |
|  | A/A | 1247(78.77%) | 639(65.74%) | Reference |  |
|  | G/A | 265(16.74%) | 273(28.09%) | 0.50(0.41,0.60) | 1.54E-12 |
|  | G/G | 22(1.39%) | 35(3.6%) | 0.32(0.19,0.55) | 3.19E-05 |
|  | Trend ^d^ |  |  |  | 1.95E-15 |
|  | Permutation ^e^ |  |  |  | <1.0E-04 |
| Step III | HLA-DRB1 amino acid Phe-67 |  |  |  |  |
|  | Non-effect/Non-effect | 745(47.06%) | 366(37.65%) | Reference |  |
|  | Effect/Non-effect | 691(43.65%) | 470(48.35%) | 0.72(0.61,0.86) | 2.32E-04 |
|  | Effect/Effect | 147(9.29%) | 136(13.99%) | 0.53(0.41,0.69) | 3.17E-06 |
|  | Trend ^d^ |  |  |  | 1.82E-07 |
|  | Permutation ^e^ |  |  |  | <1.0E-04 |
| Step IV | HLA-B amino acid Glu-45 |  |  |  |  |
|  | Non-effect/Non-effect | 1141(72.08%) | 638(65.64%) | Reference |  |
|  | Effect/Non-effect | 413(26.09%) | 291(29.94%) | 0.79(0.66,0.95) | 1.26E-02 |
|  | Effect/Effect | 29(1.83%) | 43(4.42%) | 0.38(0.23,0.61) | 6.58E-05 |
|  | Trend ^d^ |  |  |  | 2.64E-05 |
|  | Permutation ^e^ |  |  |  | <1.0E-04 |

**^a^** HLA variants identified in stepwise conditional regression. In step I, the identified effective variant was Gln-62 in HLA-A locus; in step II, the identified effective variant was rs2894207 (G); in step III, the detected variant was Phe-67 in HLA-DRB1 locus and in step IV was Glu-45 in HLA-B locus.

**^b^** Effective allele frequency in cases and controls.

**^c^** Obtained by comparing the odds of samples carrying one or two copy of effective alleles with those carrying no effective allele.

**^d^** Cochran-Armitage trend test was used to calculate the trend p values.

**^e^** Permutation was conducted 10,000 times to confirm the result.

Supplementary Table S8 LD analysis of the four identified variants in stepwise conditional analysis and the previously reported NPC associated variants. R-square and D’ values were shown in each cell. The pink cells indicated the four identified variants and the blue ones indicated the previously reported variants.

| R-square(D') | HLA-A amino acid Gln-62 | rs2894207 | HLA-DRB1 amino acid Phe-67 | HLA-B amino acid Glu-45 |
| --- | --- | --- | --- | --- |
| HLA-A amino acid Gln-62 | - | 0.022(0.26) | 0.002(0.041) | 0(0) |
| rs2894207 | 0.022(0.26) | - | 0.011(0.382) | 0.001(0.158) |
| HLA-DRB1 amino acid Phe-67 | 0.002(0.041) | 0.011(0.382) | - | 0.006(0.255) |
| HLA-B amino acid Glu-45 | 0(0) | 0.001(0.158) | 0.006(0.255) | - |
| rs2267633 | 0.358(0.764) | 0.01(0.139) | 0(0.011) | 0.001(0.119) |
| rs29230 | 0.359(0.766) | 0.011(0.141) | 0(0.011) | 0.001(0.119) |
| rs29232 | 0.268(0.779) | 0.007(0.221) | 0(0.043) | 0.044(0.485) |
| rs3129055 | 0.128(0.721) | 0.002(0.167) | 0.01(0.101) | 0.021(0.446) |
| rs9258122 | 0.128(0.721) | 0.002(0.167) | 0.01(0.101) | 0.021(0.446) |
| rs2860580 | 0.941(0.985) | 0.021(0.251) | 0.002(0.045) | 0(0.011) |
| rs3869062 | 0.994(0.681) | 0.007(0.124) | 0.003(0.07) | 0(0.027) |
| rs9260734 | 0.723(0.981) | 0.019(0.209) | 0(0.027) | 0(0.019) |
| rs5009448 | 0.527(0.753) | 0.020(0.237) | 0.001(0.034) | 0.005(0.107) |
| rs28421666 | 0.009(0.176) | 0.001(0.242) | 0.247(0.977) | 0(0.111) |
| HLA-B amino acid Leu-116 | 0.034(0.465) | 0.286(0.545) | 0.012(0.388) | 0.005(0.077) |
| HLA-C amino acid Trp-156 | 0.024(0.264) | 0.103(0.468) | 0.103(0.486) | 0.018(0.222) |

**Reference**

1. Tang, M., et al., *The principal genetic determinants for nasopharyngeal carcinoma in China involve the HLA class I antigen recognition groove.* PLoS Genet, 2012. **8**(11): p. e1003103.

2. Tse, K.P., et al., *Genome-wide association study reveals multiple nasopharyngeal carcinoma-associated loci within the HLA region at chromosome 6p21.3.* Am J Hum Genet, 2009. **85**(2): p. 194-203.

3. Bei, J.X., et al., *A genome-wide association study of nasopharyngeal carcinoma identifies three new susceptibility loci.* Nat Genet, 2010. **42**(7): p. 599-603.

4. Chin, Y.M., et al., *HLA-A SNPs and amino acid variants are associated with nasopharyngeal carcinoma in Malaysian Chinese.* Int J Cancer, 2015. **136**(3): p. 678-87.
